# Association between occupational sedentary behavior and metabolic syndrome and related diseases in males: A cross-sectional study

**DOI:** 10.1371/journal.pone.0350342

**Published:** 2026-06-26

**Authors:** Zhonglan Yin, Shaofan Weng, Dafeng Lin, Wei Zhou, Naixing Zhang

**Affiliations:** 1 Shenzhen Prevention and Treatment Center for Occupational Diseases, Shenzhen, Guangdong, China; 2 Department of Occupational and Environmental Health, State Key Laboratory of Environmental Health (Incubating), School of Public Health, Tongji Medical College, Huazhong University of Science and Technology, Wuhan, China; PRISM CRO, PAKISTAN

## Abstract

**Background:**

Prolonged occupational sedentary behavior has become a prevalent norm in workplaces, posing significant health risks. However, there is a scarcity of studies on the association between occupational sedentary behavior with metabolic syndrome (MetS) and related diseases among workers.

**Methods:**

From June 2023 to November 2024, 2,055 male bus drivers (occupational sedentary group) and sanitation workers (non-sedentary group) were enrolled by cluster sampling. Questionnaire surveys and clinical examinations were collected retrospectively. MetS was diagnosed according to the *Guidelines for the Prevention and Treatment of Diabetes Mellitus in China (2024 Edition)*. Logistic regressions and subgroup analyses were conducted to determine the associations between occupational sedentary behavior with MetS and related diseases.

**Results:**

The occupational sedentary group (1,158 participants) had higher prevalence of metabolic syndrome (24.61% vs. 22.63%), hypertriglyceridemia (51.81% vs. 36.34%), hypoalphalipoproteinemia (12.35% vs. 9.03%), and central obesity (36.62% vs. 29.43%), compared to the non-sedentary group (897 participants). In the logistic regression model, no significant correlations were observed between occupational sedentary behavior and MetS (*P* > 0.05). Compared with non-sedentary group, the sedentary group showed an increased risks of hypertriglyceridemia [OR (95% CI) = 1.77 (1.41, 2.22)], hypoalphalipoproteinemia [OR (95% CI) = 1.44 (1.01, 2.06)], and central obesity [OR (95% CI) = 1.37 (1.09, 1.74)]. Subgroup logistic regression analysis showed that among subjects aged >55 years, the associations between occupational sedentary behavior with hypertriglyceridemia and central obesity were significantly stronger (all *P* for interaction < 0.05).

**Conclusion:**

Occupational sedentary behavior in males is associated with higher prevalence of hypertriglyceridemia, hypoalphalipoproteinemia, and central obesity. Interventions to reduce sedentary behavior are needed to mitigate these risks, such as lifestyle, and organizational management strategies.

## Introduction

With the widespread adoption of digital office work, mechanized transportation, and industrial restructuring, the population engaged in static occupations has expanded rapidly, making occupational sedentary behavior a prominent characteristic of modern workstyles. Sedentary behavior refers to activities involving sitting, reclining, or lying down, with an energy expenditure ≤1.5 metabolic equivalents (METs) [1]. Surveys show that Chinese residents aged 40 years and above spend 8.8 hours daily in sedentary behavior [2], and 73.9% of employees in 79 enterprises across multiple regions report sedentary time exceeding 8 hours daily, with an average of over 9 hours per day [3]. Studies indicate that sedentary behavior, independent of physical activity levels, is associated with increased risks of chronic diseases and mortality [4,5].

Metabolic syndrome (MetS) is a cluster of metabolic abnormalities characterized by central obesity, hypertension, abnormal blood glucose, and dyslipidemia [6], and is a major risk factor for cardiovascular diseases and type 2 diabetes [7,8]. Globally, over 1 billion people are affected by MetS [9], with prevalence rates of 37.1% in U.S. adults [10], 16.0% in Africa, 21.3% in Asia, and 10.5% in Europe [11], and the incidence is rising annually [9,12]. A 2022 survey in China reported a 24.2% prevalence of MetS in adults [13]. Thus, effective intervention and management of MetS are crucial for chronic disease prevention.

Compared to leisure-time sedentary behavior, occupational sedentary behavior is often mandatory, long-term, and uncontrollable, potentially exacerbating its health impacts. Bus drivers are required to remain seated for nearly the entire work shift, with minimal mobility and limited control over their sedentary patterns [14], while sanitation workers engage in prolonged standing and physical labor throughout the workday [15]. In this study, we selected two distinct occupational groups—bus drivers as the occupational sedentary group and sanitation workers as the non-sedentary comparison group to examine the association between occupational sedentary behavior and MetS in males, aiming to explore the potential link between occupational sedentariness and MetS risk and inform future research and preventive strategies in working populations.

## Methods

### Study design and participants

**Fig 1:**
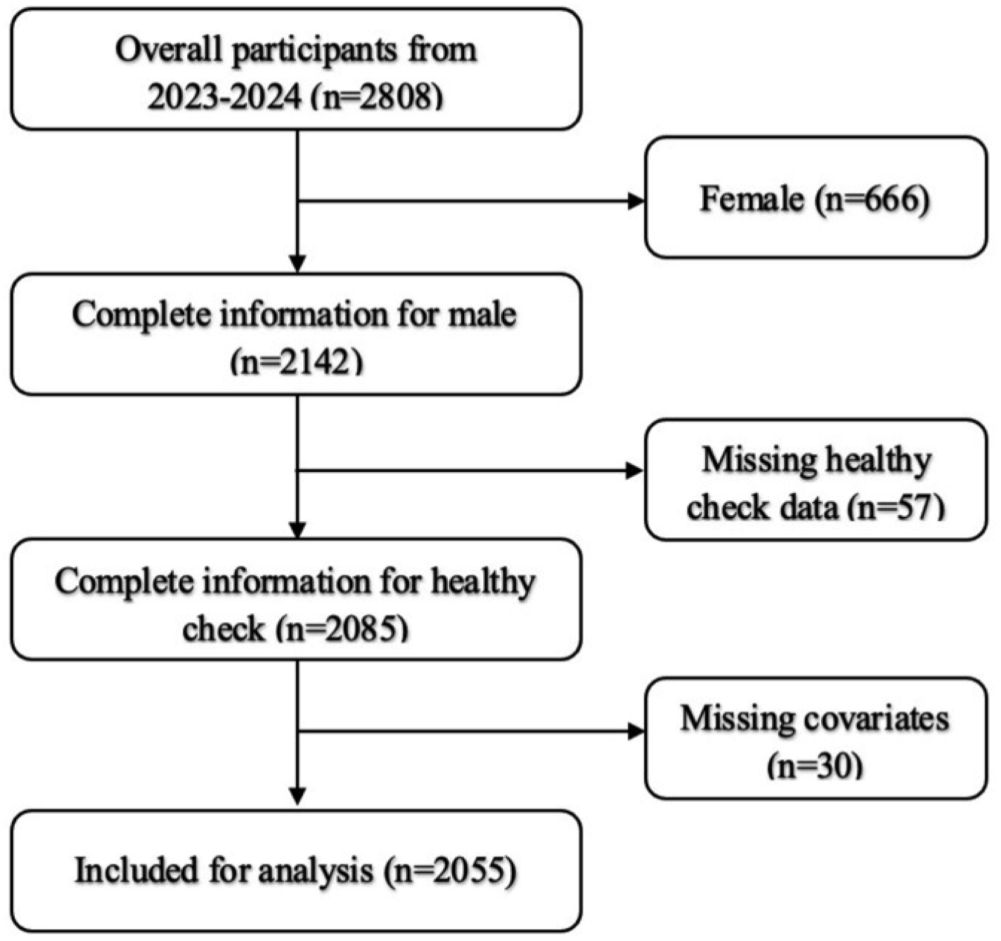
Flowchart illustrating the selection of the study population.

From June 2023 to November 2024, 2,808 participants were enrolled in Shenzhen by cluster sampling. Bus drivers were recruited from five subsidiaries of Shenzhen Bus Group, with a total of 1,159 participants. In addition, 1,649 sanitation workers were recruited from ten sanitation companies across four districts of Shenzhen, namely Luohu, Futian, Longhua and Guangming. Inclusion criteria: (1) aged ≥18 years; (2) employed in the current occupation for ≥1 year; (3) with complete data on questionnaire surveys and health examinations (Fig 1). Due to female bus drivers accounted for a relatively small proportion, all analyses in this study were restricted to male participants. Finally, after excluding 666 female, 57 participants without healthy check data, and 30 participants missing covariates, 2,055 male subjects were included in subsequent analyses. Bus drivers, with daily sedentary time >8 hours, formed the occupational sedentary group, while sanitation workers, with daily sedentary time <8 hours, formed the non-sedentary group. Notably, the study was approved by the Ethics Committee of Shenzhen Institute of Occupational Disease Prevention and Treatment (Approval No. LL2020−34). All participants in this study signed informed consent forms.

### MetS and related diseases definitions

MetS was defined according to the criteria from the *Guidelines for the Prevention and Treatment of Diabetes Mellitus* in China (2024 E*dition)* [16]. Participants having at least 3 of the following 5 risk factors were defined as having MetS: (1)central obesity: WC ≥ 90 cm for male; (2)Hyperglycemia: FBG ≥ 6.1 mmol/L, 2-hour postprandial glucose ≥7.8 mmol/L, or diagnosed diabetes; (3)Hypertension: SBP ≥ 130 mmHg, DBP ≥ 85 mmHg or diagnosed hypertension; (4)Hypertriglyceridemia: fasting triglycerides (TG) ≥1.70 mmol/L; (5) Hypoalphalipoproteinemia: fasting HDL-C < 1.04 mmol/L. MetS severity score was assessed using the Chinese age-sex-ethnicity-specific score [17]:


$$\begin{array}{cc} & \text{cMetS}=-\text{3}.\text{1436}+\text{0}.\text{0258}\times \text{WC}+\text{0}.\text{361}\times \text{TG}-\text{0}.\text{9348}\times \text{HDL}-\text{C}+\text{0}.\text{0128}\times \text{MAP}+\\ & \text{0}.\text{1224}\times \text{FBG},\text{ where MAP }=(\text{2}\times \text{DBP }+\text{ SBP})/\text{3}. \end{array}$$

### Covariates

Trained investigators collected sociodemographic, lifestyle factors, and occupational information through the structured questionnaire. The questionnaire has been applied to bus drivers, demonstrating good reliability and validity [18]. The covariates considered in this study were based on published studies [19,20]. Sociodemographic data included age, marital status (single, married, others), and education level (junior high or below, vocational/High school, college or above). Lifestyle factors included smoking status (never, current, former), and alcohol consumption (never, current, former). Work-related characteristics included duration of employment, and weekly working hours. Trained occupational health physicians collected basic anthropometric measurements [height, weight, waist circumference (WC)], body mass index (BMI), blood pressure and laboratory examination data [containing Triglyceride (TG), fasting blood glucose (FBG), high density lipoprotein cholesterol (HDL), low density lipoprotein cholesterol (LDL), and total cholesterol (TC)].

### Statistical analysis

Categorical variables were presented as frequency (percentages), and non-normal distribution continuous variables were expressed as medians (interquartile ranges). Chi-square test was used for comparison of counting data, and Mann-Whitney *U* test was applied to compare measurement data between groups. Logistic regressions, with adjustment for age, marital status, education level, smoking status, alcohol consumption, length of employment, and weekly working hours, were used to analyze associations between occupational sedentary behavior with MetS and related diseases by calculating odds ratios (ORs) and 95% confidence intervals (95% CIs), taking the non-sedentary group as the reference. Variance inflation factors (VIFs) were calculated to assess the collinearity assumption. Three models were utilized to test the above associations: model 1 was adjusted for none; model 2 was adjusted for age, marital status, education level, smoking status, and alcohol consumption; model 3 was further adjusted for duration of employment, and weekly working hours. To test for interaction effects between covariates and occupational sedentary and assess the robustness of the results, we further performed stratified logistic regression analysis to identify variables that affect the association between occupational sedentary behavior and MetS. Meanwhile, we categorized the MetS severity scores into 4 groups based on quartiles, (Q1, Q2, Q3, Q4), with Q1 as the reference, and compared Q2, Q3and Q4 with Q1 respectively. In addition, according to the number of the 5 diseases in MetS, they were divided into 0, 1, 2, and ≥3 types. With 0 types as the reference, compared 1 type, 2 types and ≥3 types with 0 types respectively. All statistical analyses were performed using R software (version 4.3.1), and *P* value less than 0.05 was considered statistically significant.

## Results

### Population characteristics

A total of 2,055 participants were enrolled in the survey with a median age of 51.0 (46.0 ~ 55.0). Among them, 1,158 participants belonged to occupational sedentary group, with a median age of 50.0 (46.0 ~ 53.0) years, duration of employment of 14.0 (8.2 ~ 18.0) years. Additionally, 897 participants belonged to non-sedentary group with a median age of 53.0 (47.0–58.0) years, duration of employment of 5.0 (2.0–10.0) years (Table 1).

**Table 1:** Baseline characteristics of occupational sedentary group and non-sedentary group.

Variables	Overall	The type of work		*P*
		Non-sedentary group	Occupational sedentary group	
Total (n)	2055	897	1158	
Age (years), n (%)				<0.001
≤ 45	452	214 (23.8)	238 (20.6)	
46 ~ 55	1117	344 (38.4)	773 (66.7)	
> 55	486	339 (37.8)	147 (12.7)	
Marital status, n (%)				<0.001
Single	83	59 (6.6)	24 (2.1)	
Married	1837	796 (88.7)	1041 (89.9)	
Others	135	42 (4.7)	93 (8.0)	
Educational level, n (%)				<0.001
Junior high or below	887	635 (70.8)	252 (21.8)	
Vocational/High school	1027	205 (22.8)	822 (71.0)	
College or above	141	57 (6.4)	84 (7.2)	
Length of work (years), n (%)				<0.001
≤ 5	633	511 (57.0)	122 (10.5)	
6 ~ 15	800	236 (26.3)	564 (48.7)	
> 15	622	150 (16.7)	472 (40.8)	
Weekly working hours, n (%)				<0.001
≤ 40	231	89 (9.9)	142 (12.3)	
41 ~ 48	748	294 (32.8)	454 (39.2)	
49 ~ 56	511	188 (21.0)	323 (27.9)	
*>* 56	565	326 (36.3)	239 (20.6)	
Smoking status, n (%)				0.293
Never	1006	425 (47.4)	581 (50.2)	
Current	772	354 (39.5)	418 (36.1)	
Former	277	118 (13.1)	159 (13.7)	
Drinking status, n (%)				0.474
Never	1811	786 (87.6)	1005 (86.8)	
Current	104	48 (5.4)	56 (4.8)	
Former	160	63 (7.0)	97 (8.4)	

### Prevalence of MetS and related diseases

The occupational sedentary group had higher prevalence of hypertriglyceridemia (51.8% vs. 36.3%), hypoalphalipoproteinemia (12.4% vs. 9.0%), and central obesity (36.6% vs. 29.4%) compared to the non-sedentary group (all *P* < 0.05). However, they had a lower prevalence of hypertension (44.1% vs. 54.6%) and hyperglycemia (23.8% vs. 29.4%) (Table 2). The occupational sedentary group also had higher comorbidity rates, with 52.1% having ≥2 diseases, and in the non-sedentary group, the comorbidity rate was 50.4% (S1 Table). Among the overall subjects, the quartile of metabolic syndrome severity score were −0.102 (Q1), 0.369 (Q2) and 0.876 (Q3). Among the occupational sedentary group, the quartiles were 0.018 (Q1), 0.458 (Q2), and 0.945 (Q3); for the non-sedentary group, the quartile were −0.249 (Q1), 0.231 (Q2), and 0.808 (Q3) (S2 Table).

**Table 2:** Prevalence of MetS and related diseases.

Outcomes	Overall	The type of work		*P*
		Non-Sedentary group	Occupational sedentary group	
Total	2055	897	1158	
MetS, n (%)				
Yes	488 (23.8)	203 (22.6)	285 (24.6)	0.295
No	1567 (76.3)	694 (77.4)	873 (75.4)	
Hypertriglyceridemia, n (%)				
Yes	926 (45.1)	326 (36.3)	600 (51.8)	**<0.001**
No	1129 (54.9)	571 (63.7)	558 (48.2)	
Hypoalphalipoproteinemia, n (%)				
Yes	224 (10.9)	81 (9.0)	143 (12.3)	**0.017**
No	1831 (89.1)	816 (91.0)	1015 (87.7)	
Central Obesity, n (%)				
Yes	688 (33.5)	264 (29.4)	424 (36.6)	**0.001**
No	1367 (66.5)	633 (70.6)	734 (63.4)	
Hyperglycemia, n (%)				
Yes	539 (26.2)	264 (29.4)	275 (23.7)	0.004
No	1516 (73.8)	633 (70.6)	883 (76.3)	
Hypertension, n (%)				
Yes	1001 (48.7)	490 (54.6)	511 (44.1)	<0.001
No	1054 (51.3)	407 (45.4)	647 (55.9)	

Categorical variables were presented as number (percentage), *P* < 0.05 presents significant difference. MetS, Metabolic Syndrome.

### Association between occupational sedentary behavior and MetS

The VIFs for all independent variables were below 2, indicating that there was no significant multicollinearity among the variables. The results showed that no significant correlations were observed between occupational sedentary behavior and MetS (*P* > 0.05) (Table 3). Compared with non-sedentary group, the occupational sedentary group had 88% higher risks of hypertriglyceridemia [OR (95% CI) =1.88 (1.58, 2.25)], 42% higher risk of hypoalphalipoproteinemia [OR (95% CI) = 1.42 (1.07, 1.90)], and 39% higher risk of central obesity [OR (95% CI) =1.39 (1.15, 1.67)]. No significant positive associations were observed between occupational sedentary behavior and hyperglycemia [OR (95% CI) = 0.75 (0.61, 0.91)] or hypertension [OR (95% CI) = 0.66 (0.55, 0.78)]. Compared with non-sedentary group, the occupational sedentary group had higher risks of MetS severity score [Q2: OR (95% CI) = 1.91 (1.49, 2.45); Q3: OR (95% CI) = 2.30 (1.79, 2.95); Q4: OR = 2.19 (1.70, 2.81)] and ≥3 diseases [OR (95% CI) = 1.31 (1.00, 1.72)]. Notably, the association of occupational sedentary behavior with hypertriglyceridemia, hypoalphalipoproteinemia, central obesity, MetS severity score and comorbidities were still significant respectively after adjustment for covariates in Model 2 and Model 3.

**Table 3:** Logistic regression analysis of the association between occupational sedentary and MetS and related diseases.

Outcomes	Model 1		Model 2		Model 3	
	OR (95% CI)	*P*	OR (95% CI)	*P*	OR (95% CI)	*P*
MetS	1.05 (0.85, 1.29)	0.681	1.17 (0.92, 1.50)	0.196	1.15 (0.89, 1.49)	0.285
Hypertriglyceridemia	1.88 (1.58, 2.25)	<0.001	1.85 (1.50, 2.29)	<0.001	1.77 (1.41, 2.22)	**<0.001**
Hypoalphalipoproteinemia	1.42 (1.07, 1.90)	0.017	1.45 (1.04, 2.03)	0.031	1.44 (1.01, 2.06)	**0.046**
Central Obesity	1.39 (1.15, 1.67)	0.001	1.52 (1.22, 1.89)	<0.001	1.37 (1.09, 1.74)	**0.009**
Hyperglycemia	0.75 (0.61, 0.91)	0.004	0.89 (0.70, 1.13)	0.339	0.83 (0.64, 1.08)	0.166
Hypertension	0.66 (0.55, 0.78)	<0.001	0.75 (0.61, 0.93)	0.007	0.77 (0.61, 0.96)	0.019
MetS severity score						
Q2 vs. Q1	1.91 (1.49, 2.45)	<0.001	1.88 (1.38, 2.57)	<0.001	1.80 (1.27, 2.56)	<0.001
Q3 vs. Q1	2.30 (1.79, 2.95)	<0.001	2.61 (1.91, 3.59)	<0.001	2.69 (1.90, 3.82)	<0.001
Q4 vs. Q1	2.19 (1.70, 2.81)	<0.001	2.20 (1.62, 2.99)	<0.001	2.01 (1.43, 2.83)	<0.001
Number of comorbid metabolic diseases						
1 vs. 0	1.23 (0.95, 1.59)	0.111	1.35 (1.00, 1.83)	0.052	1.36 (0.98, 1.89)	0.069
2 vs. 0	1.25 (0.97, 1.62)	0.090	1.49 (1.09, 2.04)	0.013	1.48 (1.05, 2.09)	0.026
≥ 3 vs. 0	1.31 (1.00, 1.72)	0.047	1.64 (1.19, 2.27)	0.003	1.54 (1.09, 2.18)	0.015

Model 1: Adjusted for none; Model 2: Adjusted for age, marital status, education level, smoking status, and drinking status; Model 3: Adjusted for age, marital status, education level, smoking status, and drinking status, length of work, and weekly working hours. MetS, Metabolic Syndrome. OR, Odds ratio. *P* < 0.05 presents significant difference.

### Subgroup analysis

**Fig 2:**
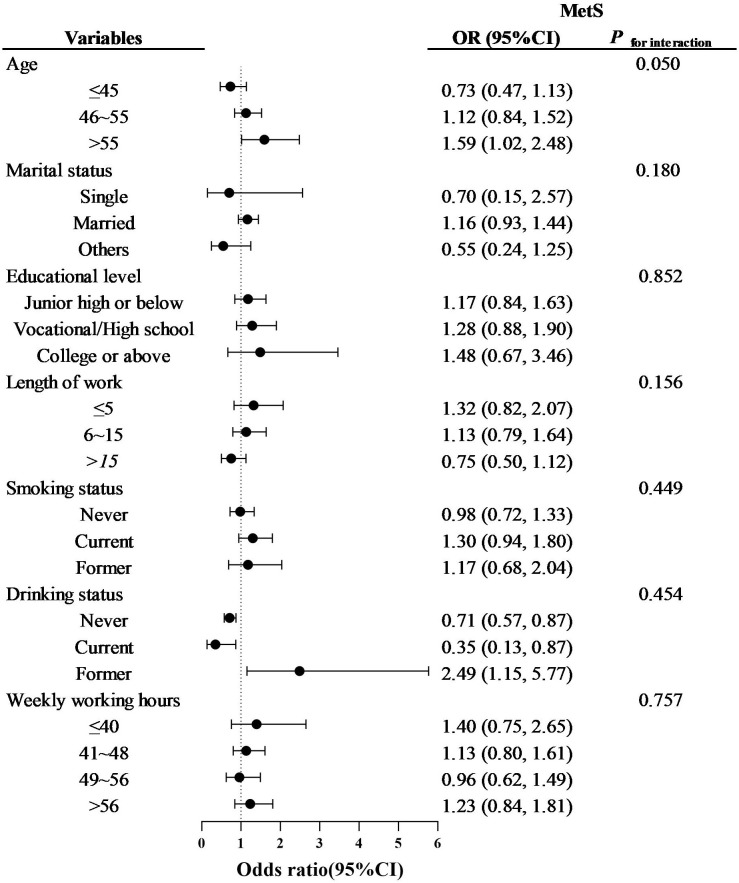
Subgroup analysis of the association between occupational sedentary and MetS.

**Fig 3:**
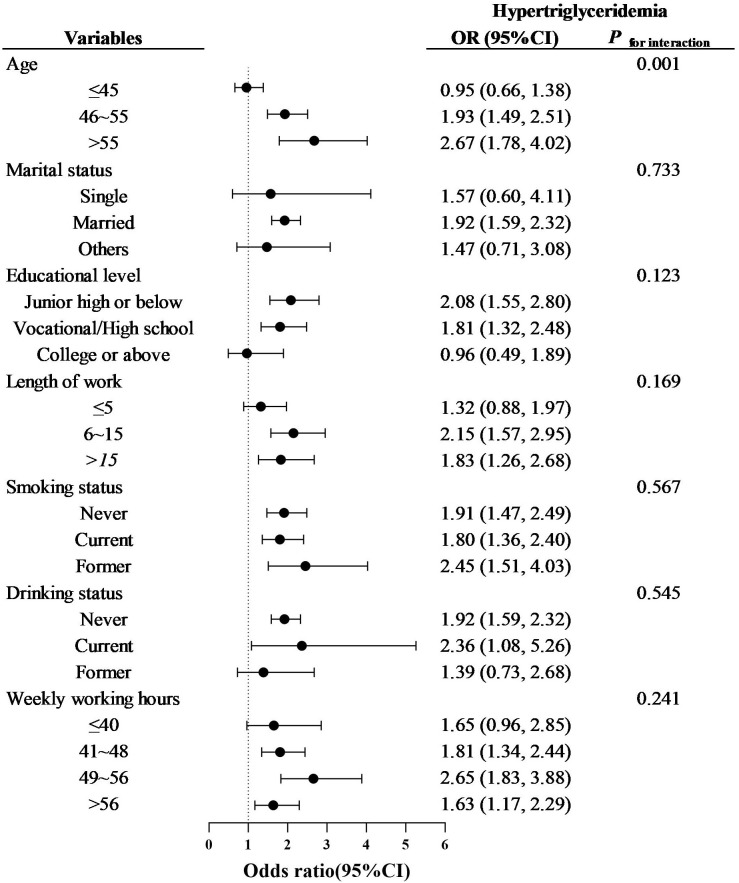
Subgroup analysis of the association between occupational sedentary and hypertriglyceridemia.

**Fig 4:**
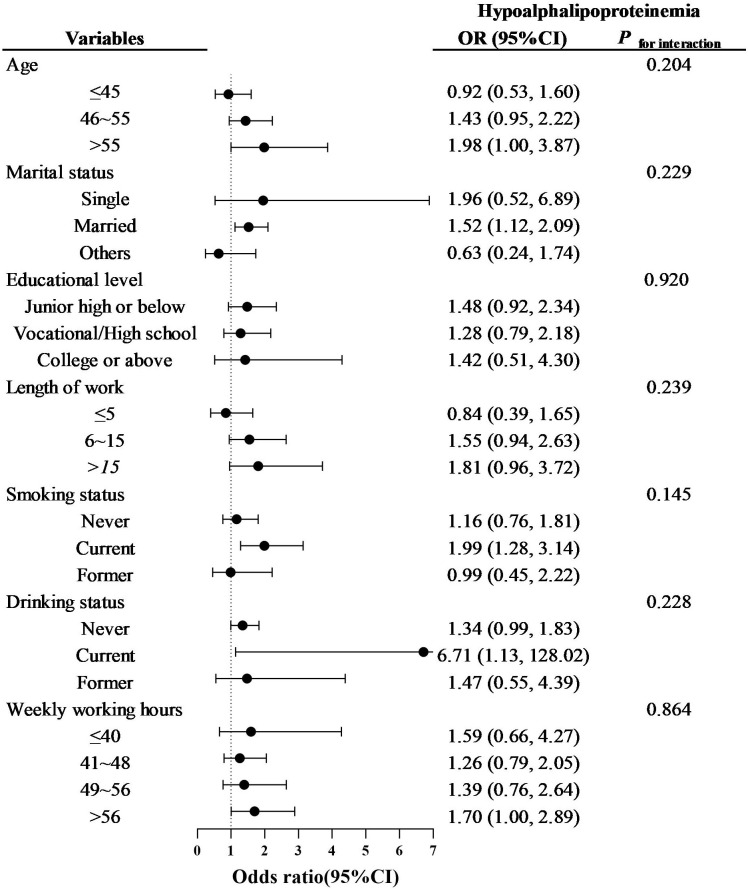
Subgroup analysis of the association between occupational sedentary and hypoalphalipoproteinemia.

**Fig 5:**
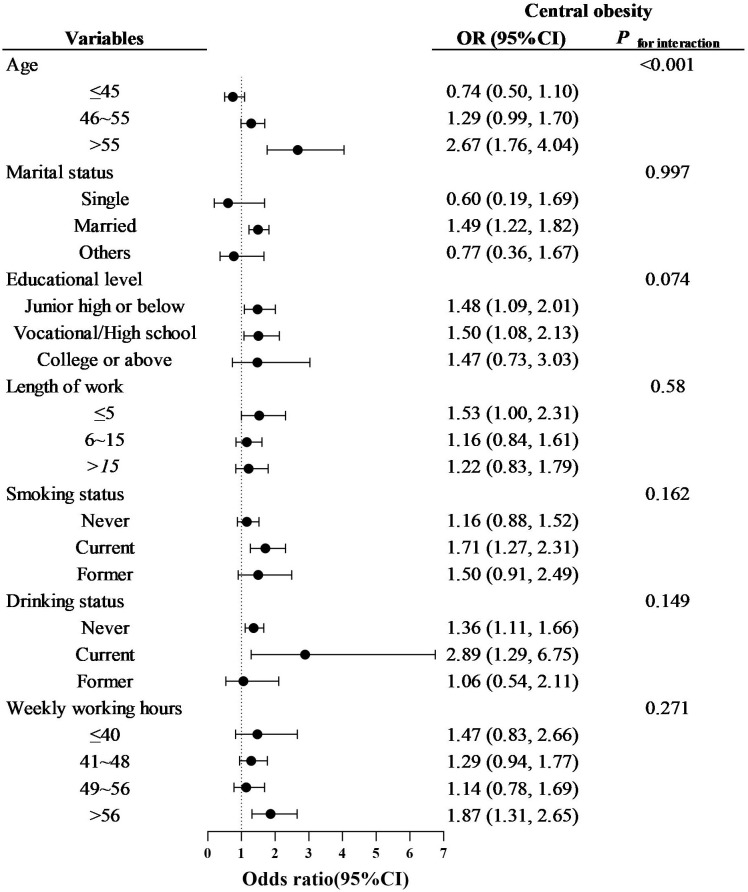
Subgroup analysis of the association between occupational sedentary and central obesity.

**Fig 6:**
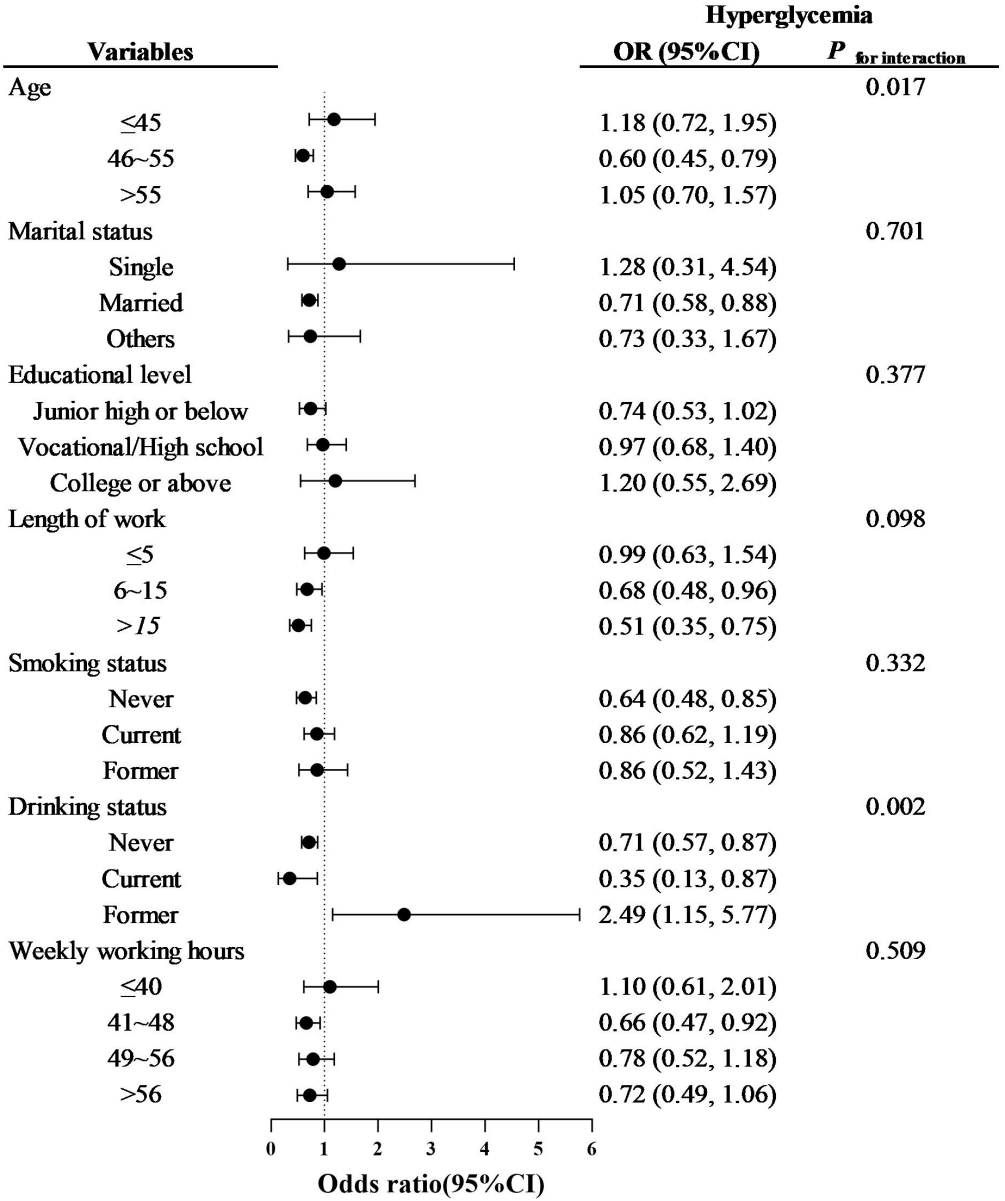
Subgroup analysis of the association between occupational sedentary and hyperglycemia.

**Fig 7:**
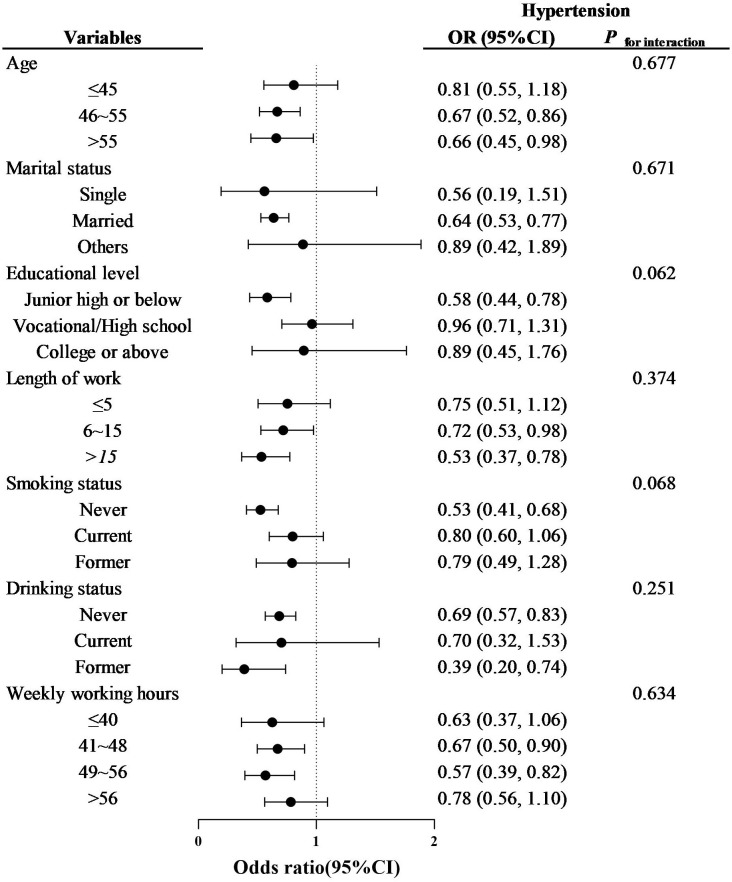
Subgroup analysis of the association between occupational sedentary and hypertension.

Stratified logistic regression analysis as well as interactive effect analysis were further performed to identify variables that may modify the association between occupational sedentary behavior and MetS and related disease (Figs 2–7). Among those aged > 55 years, the associations between occupational sedentary behavior with hypertriglyceridemia, and central obesity were enhanced (all *P* for interaction < 0.05). Occupational sedentary behavior was significantly associated with MetS among those aged > 55 years [OR (95% CI) =1.59 (1.02, 2.48), *P* for interaction = 0.050], while no significant association was observed in the younger group (≤ 45years and 46 ~ 55 years). The significant association between occupational sedentary behavior and hyperglycemia was restricted to former drinker [OR (95% CI) = 2.49 (1.15, 5.77), *P* for interaction = 0.002]. No significant modifying effects of marital status, educational level, smoking status, length of work, and weekly working hours were observed on the associations between occupational sedentary behavior and MetS or related metabolic diseases. Moreover, further stratified analyses were performed to explore the association between occupational sedentary behavior and the number of comorbid metabolic diseases (S3 Table). Occupational sedentary behavior was significantly associated with ≥ 3 related diseases among those aged > 55 years [OR (95% CI) = 2.40 (1.28, 4.66), *P* for interaction = 0.043]. The associations between occupational sedentary behavior with 2 or ≥3 related diseases were enhanced among the former smoker (*P* for interaction were 0.011 and 0.023, respectively).

## Discussion

Occupational sedentary behavior has emerged as a critical risk factor for MetS. In this study, the overall MetS prevalence was 23.75%, with the sedentary group (24.61%) slightly exceeding the reported prevalence among Chinese adults (24.20%) [13] and being higher than rates in Guangdong (20.68%) [21] and Shenzhen (17.70%) [22]. Specifically, the sedentary group showed significantly higher rates of hypertriglyceridemia, hypoalphalipoproteinemia, and central obesity. The findings of Du Wenhui, et al. demonstrated a strong association between sedentary behaviors and dyslipidemia among scientific and technological workers [23], which aligns with our results. An analysis of a health examination cohort in southwestern China further confirmed that sedentary behavior was significantly associated with abnormal triglyceride and HDL levels [24]. A regional study among hyperglycemic patients in Pakistan found that dyslipidemia was highly prevalent (95%), and the most prevalent lipid abnormality was high LDL (88%) followed by low HDL (71.5%), reflecting a similar clustering of metabolic abnormalities [25].

Mechanistically, sedentary behavior reduces skeletal muscle activity, lowering lipoprotein lipase (LPL) activity and may impair tissue uptake of triglycerides, contributing to elevated triglyceride levels and decreased HDL-C [26,27]. Additionally, reduced skeletal muscle contraction decreases interleukin-6 (IL-6) secretion [28], IL-6 is involved in insulin-stimulated glucose uptake, lipolysis, fatty acid oxidation, and energy expenditure [29]. Occupational sedentary behavior also increases free fatty acid release, exacerbating lipid metabolism disorders [30]. Energy expenditure reduction during sedentary behavior also promotes visceral fat accumulation and central obesity [31]. These findings suggest that occupational sedentary behavior may be a risk factor of MetS and related diseases. As outlined in recent work on the molecular mechanisms of diabetes mellitus, chronic hyperglycemia and dyslipidemia are not isolated abnormalities but key pathophysiological drivers of progressive pancreatic β-cell dysfunction, insulin resistance, and systemic metabolic disturbances [32]. These molecular pathways highlight the importance of addressing sedentary behavior as a modifiable risk factor to mitigate long-term metabolic complications in high-risk working populations.

Notably, the sedentary group showed lower prevalence rates of hypertension and hyperglycemia than the no-sedentary group, possibly attributable to workplace regulations. Bus drivers undergo regular blood pressure monitoring, with high-risk individuals required to measure their blood pressure daily before driving; those failing to meet standards are prohibited from operating vehicles [33]. In contrast, sanitation workers, who have less aware of their blood pressure status and take fewer preventive measures, show higher hypertension detection rates [34,35]. Additionally, given higher exposure to dust and environmental pollutants, sanitation workers may face increased risks of hyperglycemia [36].

A cross-sectional study of 5,739 adults found that prolonged sitting was associated with more severe MetS [37]. A review of multiple prospective cohort studies further confirmed sedentary behavior as a significant risk factor for MetS progression [38]. Our results also showed that occupational sedentary group had higher severity of MetS, reinforcing the role of sedentary behavior in the progression of MetS. A cohort study of 360,047 participants from the UK Biobank found that individuals sitting >6 hours/day had a 26% higher risk of developing 12 chronic diseases compared to those sitting ≤2 hours/day [39]. In our study, the occupational sedentary group exhibited significantly higher comorbidity rates, prevalence of ≥3 diseases, and average number of diseases than the no-sedentary group. Notably, the risk of having ≥3 diseases [OR (95% CI) = 1.31 (1.00, 1.72)] was higher than the risk of no disease, suggesting that occupational sedentariness not only associated with individual metabolic disorders but also promotes multimorbidity.

In addition, subgroup analysis showed that the association between occupational sedentariness with hypertriglyceridemia, hypoalphalipoproteinemia, and central obesity were enhanced among those aged >55 years. Previous study has showed that aging is accompanied by increased oxidative stress and chronic low-grade inflammation, and these processes accelerate adipose tissue dysfunction, visceral fat accumulation, and insulin resistance, thereby amplifying the risk of metabolic disorders [40]. Among these participants, limiting daily sedentary time is vital for preventing and controlling MetS and related diseases risk.

In the present study, we investigated not only the potential role of occupational sedentary on MetS and related diseases, but also MetS severity score and comorbid metabolic diseases. The results showed that age, smoking, drinking, and working >56 hours/week may modify the effect in a risk-increasing manner. Nevertheless, our study has several limitations. Firstly, the cross-sectional study design makes the causal relationship difficult to explain, and the generalizability of our results may be affected. Secondly, the variables of questionnaire were self-reported, which may introduce self-report and recall bias into the analysis. Thirdly, despite adjustments for confounding factors, residual confounding may persist due to inherent differences in socioeconomic status, work pressure, and lifestyle factors (e.g., diet, non-occupational physical activity) across occupational groups. Fourthly, the comparison between bus drivers and sanitation workers may be affected by the Healthy Worker Survivor Effect, which may artificially lower the prevalence of hypertension and Hyperglycemia in the remaining driver cohort. Future studies should further explore the prevalence of hypertension and hyperglycemia in other occupational sedentary populations. Additionally, our study categorized participants as occupational sedentary group and no-sedentary group solely based on their occupational types, future studies should use objective measurements of sedentary time. Due to the substantial differences in work environments, occupational stress, and environmental exposures between the two occupational groups, the study only identifies occupational sedentariness as a potential contributing factor, and future studies are needed to further validate these findings.

## Conclusion

Occupational sedentary behavior is associated with hypertriglyceridemia, hypoalphalipoproteinemia, and central obesity among males, with interactions involving age. To mitigate these risks, comprehensive interventions aimed at reducing sedentary behavior should be implemented in conjunction with lifestyle adjustments, and organizational management systems.

## Supporting information

S1 Table Baseline characteristics of study participants by number of comorbid metabolic diseases. (DOC)

S2 Table Baseline characteristics by MetS severity score quartiles. (DOC)

S3 Table Subgroup analysis of the association between occupational sedentary behavior and number of comorbid metabolic diseases. (DOC)
