# Supplementary material for: Association between occupational sedentary behavior and metabolic syndrome and related diseases in males: A cross-sectional study
Source: PLoS One. 2026 Jun 26;21(6):e0350342. doi: 10.1371/journal.pone.0350342 (PMC13308799; doi:10.1371/journal.pone.0350342)
Supplement: S1 Table — (DOC) [file pone.0350342.s001.doc]

| **S1 Table. Baseline characteristics of study participants by number of comorbid metabolic diseases** | | | | | | | |
| --- | --- | --- | --- | --- | --- | --- | --- |
| Variables | Overall | Number of comorbid metabolic diseases | | | | **Mean number of diseases** | **Comorbidity rate** (%) |
|  |  | 0 | 1 | 2 | ≥3 |  |  |
| Total | 2055 | 387 (18.8) | 598 (29.1) | 582 (28.3) | 488 (23.8) | 1.64 | 52.1 |
| Age (years), n (%) |  |  |  |  |  |  |  |
| ≤45 | 452 | 103 (22.8) | 128 (28.3) | 116 (25.7) | 105 (23.2) | 1.56 | 48.9 |
| 46~55 | 1117 | 196 (17.6) | 325 (29.1) | 323 (28.9) | 273 (24.4) | 1.69 | 53.4 |
| >55 | 486 | 88 (18.1) | 145 (29.8) | 143 (29.4) | 110 (22.6) | 1.62 | 52.1 |
| Ethnicity, n (%) |  |  |  |  |  |  |  |
| Han | 1971 | 370 (18.8) | 585 (29.7) | 551 (28.0) | 465 (23.6) | 1.64 | 51.6 |
| Others | 84 | 17 (20.2) | 13 (15.5) | 31 (36.9) | 23 (27.4) | 1.83 | 64.3 |
| Marital status, n (%) |  |  |  |  |  |  |  |
| Single | 83 | 21 (25.3) | 22 (26.5) | 27 (32.5) | 13 (15.7) | 1.42 | 48.2 |
| Married | 1837 | 339 (18.5) | 538 (29.3) | 519 (28.3) | 441 (24.0) | 1.65 | 52.3 |
| Others | 135 | 27 (20.0) | 38 (28.2) | 36 (26.7) | 34 (25.2) | 1.66 | 51.9 |
| Educational level, n (%) |  |  |  |  |  |  |  |
| Junior high or below | 887 | 160 (18.0) | 256 (28.9) | 251 (28.3) | 220 (24.8) | 1.67 | 53.1 |
| Vocational/High school | 1027 | 202 (19.7) | 300 (29.2) | 290 (28.2) | 235 (22.9) | 1.62 | 51.1 |
| College or above | 141 | 25 (17.7) | 42 (29.8) | 41 (29.1) | 33 (23.4) | 1.65 | 52.5 |
| The type of work, n (%) |  |  |  |  |  |  |  |
| No-sedentary group | 897 | 187 (20.9) | 258 (28.8) | 249 (27.8) | 203 (22.6) | 1.59 | 50.4 |
| Occupational sedentary group | 1158 | 200 (17.3) | 340 (29.4) | 333 (28.8) | 285 (24.6) | 1.69 | 53.4 |
| Length of work (years), n (%) |  |  |  |  |  |  |  |
| ≤5 | 633 | 131 (20.7) | 189 (29.9) | 177 (28.0) | 136 (21.5) | 1.56 | 49.5 |
| 6 ~15 | 800 | 153 (19.1) | 235 (29.4) | 227 (28.4) | 185 (23.1) | 1.63 | 51.5 |
| *>15* | 622 | 103 (16.6) | 174 (28.0) | 178 (28.6) | 167 (26.9) | 1.75 | 55.5 |
| Weekly working hours, n (%) |  |  |  |  |  |  |  |
| ≤40 | 231 | 51 (22.1) | 57 (24.7) | 65 (28.1) | 58 (25.1) | 1.62 | 53.3 |
| 41~48 | 748 | 132 (17.7) | 231 (30.9) | 207 (27.7) | 178 (23.8) | 1.64 | 51.5 |
| 49~56 | 511 | 96 (18.8) | 161 (31.5) | 145 (28.4) | 109 (21.3) | 1.61 | 49.7 |
| *>*56 | 565 | 108 (19.1) | 149 (26.4) | 165 (29.2) | 143 (25.3) | 1.69 | 54.5 |
| Smoking status, n (%) |  |  |  |  |  |  |  |
| Never | 1006 | 197 (19.6) | 322 (32.0) | 281 (27.9) | 206 (20.5) | 1.56 | 48.4 |
| Current | 772 | 152 (19.7) | 189 (24.5) | 222 (28.8) | 209 (27.1) | 1.72 | 55.8 |
| Former | 277 | 38 (13.7) | 87 (31.4) | 79 (28.5) | 73 (26.4) | 1.74 | 54.9 |
| Drinking status, n (%) |  |  |  |  |  |  |  |
| Never | 1811 | 355 (19.6) | 535 (29.5) | 494 (27.3) | 427 (23.6) | 1.62 | 50.9 |
| Current | 104 | 13 (12.5) | 27 (26.0) | 36 (34.6) | 28 (26.9) | 1.80 | 61.5 |
| Former | 160 | 39 (24.4) | 36 (22.5) | 52 (32.5) | 33 (20.6) | 1.58 | 53.1 |
| Categorical variables were presented as number (percentage), *P* < 0.05 presents significant difference. | | | | | | | |
