# Supplementary material for: Association between occupational sedentary behavior and metabolic syndrome and related diseases in males: A cross-sectional study
Source: PLoS One. 2026 Jun 26;21(6):e0350342. doi: 10.1371/journal.pone.0350342 (PMC13308799; doi:10.1371/journal.pone.0350342)
Supplement: S2 Table — (DOC) [file pone.0350342.s002.doc]

| **S2 Table. Baseline characteristics by MetS severity score quartiles** | | |
| --- | --- | --- |
| Variables | MetS severity score | *P* |
| Total | 0.369 (-0.102~0.876) |  |
| Age (years) |  | <0.001 |
| ≤45 | 0.394 (-0.102~0.959) |  |
| 46~55 | 0.408 (-0.048~0.920) |  |
| >55 | 0.249 (-0.209~0.749) |  |
| Ethnicity |  | 0.321 |
| Han | 0.367 (-0.102~0.874) |  |
| Others | 0.497 (-0.108~0.906) |  |
| Marital status |  | 0.323 |
| Single | 0.236 (-0.223~0.866) |  |
| Married | 0.383 (-0.102~0.880) |  |
| Others | 0.327 (-0.010~0.836) |  |
| Educational level |  | 0.020 |
| Junior high or below | 0.318 (-0.162~0.847) |  |
| Vocational/High school | 0.395 (-0.055~0.899) |  |
| College or above | 0.450 (0.018~0.866) |  |
| The type of work |  | <0.001 |
| No-sedentary group | 0.231(-0.249~0.808) |  |
| Occupational sedentary group | 0.458 (0.018~0.945) |  |
| Length of work (years) |  | <0.001 |
| ≤5 | 0.251 (-0.195~0.799) |  |
| 6 ~15 | 0.385 (-0.072~0.899) |  |
| *>15* | 0.439 (-0.005~0.949) |  |
| Weekly working hours |  | 0.709 |
| ≤40 | 0.354 (-0.074~0.895) |  |
| 41~48 | 0.406 (-0.058~0.858) |  |
| 49~56 | 0.323 (-0.136~0.846) |  |
| *>*56 | 0.368 (-0.121~0.909) |  |
| Smoking status |  | <0.001 |
| Never | 0.295 (-0.142~0.780) |  |
| Current | 0.470 (-0.0719~1.04) |  |
| Former | 0.405 (0.033~0.886) |  |
| Drinking status |  | 0.212 |
| Never | 0.367 (-0.115~0.875) |  |
| Current | 0.531 (0.037~1.120) |  |
| Former | 0.368 (-0.068~0.807) |  |
| Continuous variables were presented as median (P_25_-P_75_), *P* < 0.05 presents significant difference. | | |
