# Supplementary material for: Association between occupational sedentary behavior and metabolic syndrome and related diseases in males: A cross-sectional study
Source: PLoS One. 2026 Jun 26;21(6):e0350342. doi: 10.1371/journal.pone.0350342 (PMC13308799; doi:10.1371/journal.pone.0350342)
Supplement: S3 Table — (DOC) [file pone.0350342.s003.doc]

| **S3 Table. Subgroup analysis of the association between occupational sedentary behavior and number of comorbid metabolic diseases** | | | | | | | | |
| --- | --- | --- | --- | --- | --- | --- | --- | --- |
| Variables | 1vs 0 | |  | 2 vs 0 | |  | ≥3 vs 0 | |
|  | OR (95% CI) | *P* |  | OR (95% CI) | *P* |  | OR (95% CI) | *P* |
| Age |  |  |  |  |  |  |  |  |
| ≤45 | 1.38 (0.82, 2.33) | 0.229 |  | 1.01 (0.59, 1.72) | 0.968 |  | 0.83 (0.48, 1.42) | 0.490 |
| 46~55 | 1.20 (0.82, 1.76) | 0.338 |  | 1.16 (0.79, 1.69) | 0.446 |  | 1.28 (0.86, 1.89) | 0.227 |
| >55 | 1.33 (0.71, 2.57) | 0.378 |  | 2.09 (1.14, 3.97) | 0.020 |  | 2.40 (1.28, 4.66) | 0.008 |
| *P* _for interaction_ | 0.907 |  |  | 0.174 |  |  | 0.043 |  |
| Marital status |  |  |  |  |  |  |  |  |
| Single | 0.44 (0.10, 1.78) | 0.261 |  | 1.18 (0.36, 4.01) | 0.79 |  | 0.60 (0.11, 2.76) | 0.526 |
| Married | 1.21 (0.92, 1.59) | 0.176 |  | 1.22 (0.92, 1.60) | 0.165 |  | 1.34 (1.01, 1.78) | 0.045 |
| Others | 2.58 (0.87, 7.95) | 0.090 |  | 2.06 (0.71, 6.19) | 0.187 |  | 0.98 (0.35, 2.75) | 0.973 |
| P _for interaction_ | 0.144 |  |  | 0.641 |  |  | 0.528 |  |
| Educational level |  |  |  |  |  |  |  |  |
| Junior high or below | 1.62 (1.03, 2.59) | 0.038 |  | 1.35 (0.85, 2.18) | 0.203 |  | 1.60 (1.00, 2.58) | 0.051 |
| Vocational/High school | 1.28 (0.84, 1.94) | 0.251 |  | 1.71 (1.10, 2.65) | 0.018 |  | 1.69 (1.07, 2.70) | 0.027 |
| College or above | 0.89 (0.32, 2.42) | 0.819 |  | 0.85 (0.31, 2.33) | 0.756 |  | 1.33 (0.45, 3.96) | 0.601 |
| P _for interaction_ | 0.509 |  |  | 0.433 |  |  | 0.923 |  |
| Length of work |  |  |  |  |  |  |  |  |
| ≤5 | 1.09 (0.62, 1.94) | 0.779 |  | 0.91 (0.50, 1.66) | 0.754 |  | 1.32 (0.73, 2.41) | 0.367 |
| 6~15 | 1.58 (1.02, 2.44) | 0.042 |  | 1.41 (0.91, 2.18) | 0.124 |  | 1.52 (0.96, 2.41) | 0.076 |
| >15 | 0.93 (0.52, 1.63) | 0.791 |  | 1.20 (0.66, 2.14) | 0.545 |  | 0.78 (0.44, 1.36) | 0.381 |
| P _for interaction_ | 0.305 |  |  | 0.409 |  |  | 0.182 |  |
| Smoking status |  |  |  |  |  |  |  |  |
| Never | 0.97 (0.68, 1.39) | 0.876 |  | 0.87 (0.60, 1.25) | 0.444 |  | 0.92 (0.62, 1.36) | 0.668 |
| Current | 1.30 (0.85, 1.99) | 0.236 |  | 1.64 (1.08, 2.49) | 0.020 |  | 1.72 (1.13, 2.63) | 0.012 |
| Former | 2.67 (1.23, 5.99) | 0.014 |  | 2.65 (1.21, 6.02) | 0.017 |  | 2.60 (1.17, 5.95) | 0.021 |
| P _for interaction_ | 0.063 |  |  | 0.011 |  |  | 0.023 |  |
| Drinking status |  |  |  |  |  |  |  |  |
| Never | 1.36 (1.04, 1.80) | 0.027 |  | 1.25 (0.95, 1.66) | 0.111 |  | 1.36 (1.02, 1.82) | 0.036 |
| Current | 0.50 (0.13, 1.92) | 0.317 |  | 1.20 (0.33, 4.34) | 0.779 |  | 1.54 (0.40, 5.97) | 0.525 |
| Former | 0.70 (0.28, 1.75) | 0.444 |  | 1.29 (0.54, 3.08) | 0.569 |  | 0.85 (0.33, 2.19) | 0.733 |
| P _for interaction_ | 0.157 |  |  | 0.996 |  |  | 0.626 |  |
| Weekly working hours |  |  |  |  |  |  |  |  |
| ≤40 | 0.53 (0.24, 1.14) | 0.105 |  | 1.00 (0.46, 2.14) | 0.992 |  | 1.12 (0.50, 2.49) | 0.78 |
| 41~48 | 1.11 (0.71, 1.71) | 0.656 |  | 0.99 (0.64, 1.55) | 0.978 |  | 1.18 (0.74, 1.86) | 0.494 |
| 49~56 | 1.48 (0.88, 2.48) | 0.136 |  | 1.69 (0.99, 2.89) | 0.053 |  | 1.35 (0.77, 2.36) | 0.298 |
| >56 | 1.59 (0.95, 2.68) | 0.077 |  | 1.51 (0.92, 2.52) | 0.109 |  | 1.71 (1.03, 2.89) | 0.041 |
| P _for interaction_ | 0.095 |  |  | 0.371 |  |  | 0.710 |  |
| **Subgroup** analyses were conducted by age, marital status, education level, length of work, smoking status, drinking status, and weekly working hours, *P* < 0.05 presents significant difference. | | | | | | | | |
