# Supplementary material for: Association between occupational sedentary behavior and metabolic syndrome and related diseases in males: A cross-sectional study
Source: PLoS One. 2026 Jun 26;21(6):e0350342. doi: 10.1371/journal.pone.0350342 (PMC13308799; doi:10.1371/journal.pone.0350342)
Supplement: Attachment — Submitted filename: Comments to Author.pdf [file pone.0350342.s004.pdf]

Comments to Author

Title & Abstract

The title is clear and reflects the main focus of the study. However, consider specifying the study design (cross-sectional) and population to improve transparency and indexing.

The abstract summarizes the objectives and findings adequately. However:

- Add a brief statement regarding major methodological limitations, particularly the use of self-reported outcomes and cross-sectional design.
- Clarify whether the adjusted models included all confounders described in the Methods section.
- Ensure that all reported statistical estimates (OR, AOR, CI, and p-values) match the final tables.
- The conclusion should avoid causal interpretation and emphasize associations.

Introduction

The background is comprehensive, but some global epidemiology is repetitive. Consider shortening general IBD information and focusing more on regional relevance and public health significance.

The rationale is present but should more clearly identify the knowledge gap in the target population. Explain why this study is needed locally and how it contributes to existing literature.

The study objectives are stated; however, specify:

- Primary exposure variables
- Hypothesis-driven modifiable risk factors
- Expected public health implications

Materials & Methods

Study Design and Setting:

The cross-sectional design is appropriate for exploratory risk factor analysis. However, explicitly justify why this design was chosen and acknowledge its limitations.

Participants and Eligibility

Clarify how eligibility criteria were verified.

If residence duration or disease status was self reported, this should be clearly stated.

Sampling and Recruitment

Provide detailed information on recruitment:

• Percentage recruited via online platforms

• Percentage from patient support groups

• Any offline recruitment

Data Collection Instrument

The questionnaire should include:

• Information on validation

• Language adaptation and pilot testing

• Availability in supplementary files

If validation was not performed, this should be acknowledged.

Variables and Measurements

Clearly define:

• Exposure categories

• Reference groups

• Cut-off values

• How missing data were handled

Avoid ambiguity in classification.

Sample Size

The current justification appears descriptive. Consider revising based on:

• Logistic regression assumptions

• Expected effect size

• Number of predictors

Statistical Analysis

This section requires further clarity:

• Specify the primary regression model

• Describe adjustment strategy

• Explain model selection criteria

• Clarify variable coding and reference groups

• Include assessment of multicollinearity

• Add model diagnostics (calibration and discrimination)

Results

Ensure all baseline characteristics are reported consistently as n/N (%).

Recheck percentages and totals to avoid discrepancies.

Tables:

Tables should be recreated directly from statistical software outputs to avoid typographical errors.

Include:

• Unadjusted and adjusted analyses

• Supplementary tables showing stepwise adjustment

Clarify interpretation of significant findings. Avoid overstating the importance of weak or borderline associations.

Discussion

The discussion is well structured but requires stronger linkage to global literature and regional implications.

Causal language should be minimized. The cross-sectional design prevents temporal inference.

Examples:

• Diet and lifestyle factors may be consequences rather than causes.

The biological plausibility section is good but could be expanded:

• Microbiome

• Environmental exposures

• Stress and immune regulation

Limitations need to be strengthened:

• Selection bias

• Recall bias

• Self-reported diagnosis

• Residual confounding

• Lack of objective biomarkers

Future research directions should include:

• Cohort studies

• Case-control designs

• Objective diagnostic tools

• Biomarker-based studies

Conclusion

The conclusion is generally appropriate but should:

• Emphasize associations

• Avoid clinical or policy recommendations

• Highlight need for longitudinal evidence
