# Supplementary material for: Association between occupational sedentary behavior and metabolic syndrome and related diseases in males: A cross-sectional study
Source: PLoS One. 2026 Jun 26;21(6):e0350342. doi: 10.1371/journal.pone.0350342 (PMC13308799; doi:10.1371/journal.pone.0350342)
Supplement: Attachment — Submitted filename: Recommendation.pdf [file pone.0350342.s005.pdf]

While the study utilizes a robust sample size of 2,055 male workers, there are critical flaws in how the data is interpreted and reported specifically a direct contradiction between the statistical results and the abstract's conclusions.

1. The abstract concludes that "Occupational sedentary behavior in males is associated with higher prevalence of metabolic syndrome...". However, this is demonstrably false based on the study's own data. In the results section of the abstract, the authors report an Odds Ratio for MetS of 1.05 (95%CI: 0.85-1.29). Since the 95% confidence interval crosses 1.0, this is not a statistically significant finding.
2. The abstract must be rewritten to accurately reflect that occupational sedentary behavior was associated with *specific components* of metabolic syndrome (hypertriglyceridemia, hypoalphalipoproteinemia, and central obesity), but not overall MetS itself.
3. The introduction effectively sets up the premise, noting that occupational sedentary behavior is distinct from leisure-time sedentary behavior because it is mandatory and uncontrollable.
4. The authors should briefly introduce why bus drivers and sanitation workers were specifically chosen as the proxies for sedentary and non-sedentary groups, as these two professions entail vastly different environmental and stress exposures.
5. The fundamental flaw in the study design is comparing bus drivers (sedentary) to sanitation workers (non-sedentary). These two groups differ in many ways beyond just sitting time. The authors must acknowledge that bus driving involves high psychological stress, shift work, and vibration, while sanitation work involves heavy physical exertion and high exposure to outdoor pollutants.
6. While the authors adjusted for age, ethnicity, marital status, education level, smoking status, drinking status, length of work, and weekly working hours, they failed to measure or adjust for two massive confounders:
7. Caloric intake and dietary habits are primary drivers of MetS.
8. A sanitation worker might rest after work, while a bus driver might exercise. Without adjusting for non-occupational physical activity, isolating the effect of *occupational* sedentariness is highly problematic.
9. The data shows that the sedentary group had a *lower* prevalence of hypertension (44.1% vs. 54.6%) and hyperglycemia (23.8% vs. 29.4%) compared to the non-sedentary group. In the main text, the authors write: Interestingly, despite no significant difference was observed between the groups (P=0.681), the trend of a higher prevalence of MetS in occupational

sedentary group.. Reporting a non-significant result as a trend of higher prevalence is statistically inappropriate and must be removed.

10. Table 3 confirms that MetS is not significantly associated with sedentary behavior across Model 1 (p=0.681), Model 2 (p=0.196), or Model 3 (p=0.285). The manuscript must be revised overall to remove claims that a direct association with overall MetS was found.

11. The authors attempt to explain the lower rates of hypertension and hyperglycemia in bus drivers by citing workplace regulations namely, that bus drivers undergo strict blood pressure monitoring and those failing to meet standards are prohibited from driving. This is a textbook example of the Healthy Worker Survivor Effect. Because sick bus drivers are removed from the population, the remaining drivers appear artificially healthier in these metrics. This severely limits the validity of comparing these two specific cohorts and must be explicitly detailed as a major limitation.

12. The discussion regarding skeletal muscle activity, lipoprotein lipase (LPL), and interleukin-6 (IL-6) is well-supported and provides a good biological mechanism for the lipid abnormalities observed. The limitations section currently notes the cross-sectional design and self-reported questionnaire variables. It must be expanded to explicitly state the unmeasured confounders (diet, non-work physical activity) and the immense baseline differences between the specific occupations chosen.

There are a few papers that align with the physiological and metabolic aspects of the manuscript. We suggest to appropriately integrate them:

- Sarfraz, M., Sajid, S., & Ashraf, M. A. (2016). Prevalence and pattern of dyslipidemia in hyperglycemic patients and its associated factors among Pakistani population. *Saudi journal of biological sciences*, *23*(6), 761-766.

Where: In the **Discussion** section. The authors discuss the prevalence of hypertriglyceridemia and hyperglycemia in their cohorts. You use this paper to contextualize the regional prevalence of dyslipidemia and how it clusters with hyperglycemia in Asian populations.

- Abbas, G., Salman, A., Rahman, S. U., Ateeq, M. K., Usman, M., Sajid, S., ... & Younas, T. (2017). Aging mechanisms: linking oxidative stress, obesity and inflammation. *Matrix Sci. Med*, *1*(1), 30-33.

Where: In the **Discussion** section regarding the subgroup analysis. The authors note a significant interaction between age (>55 years) and central obesity. You use this paper to help

them explain the biological mechanisms, specifically how aging and inflammation exacerbate obesity-related metabolic dysfunction

- Toor, I. F., Sajid, M., & Sajid, H. U. (2024). Molecular Mechanisms of Diabetes Mellitus. *Fundamentals of Cellular and Molecular Biology*, 156-176.

Where: In the **Introduction** or **Discussion**. The authors should expand on the long-term consequences of the hyperglycemia and metabolic syndrome observed in their study.

These recent publications could serve as up-to-date references on the molecular progression and management of diabetes.
