# Supplementary material for: Association between occupational sedentary behavior and metabolic syndrome and related diseases in males: A cross-sectional study
Source: PLoS One. 2026 Jun 26;21(6):e0350342. doi: 10.1371/journal.pone.0350342 (PMC13308799; doi:10.1371/journal.pone.0350342)
Supplement: Attachment — Submitted filename: Response to Reviewers.docx [file pone.0350342.s007.docx]

Thanks for your letter and for the reviewer’s comments concerning our manuscript entitled “Association between occupational sedentary behavior and metabolic syndrome and related diseases in males: A cross-sectional study” (Manuscript ID: PONE-D-25-67473). Those comments are valuable and helpful for revising and improving our paper. We have studied all comments carefully and have made conscientious corrections.

Revised portions are marked in red in the paper. The main corrections in the paper and the responses to the reviewers’ comments are as follows:

Reviewer #1

**Comment 1:** The association between occupational sedentary behavior and metabolic syndrome was not statistically significant; therefore, it is recommended that this relationship be revised accordingly in both the abstract and the conclusion.

**Response:** We sincerely appreciate your comment. We have revised the description of the relationship between occupational sedentary behavior and metabolic syndrome to “no statistically significant association” in the abstract (lines 39-41) and have also revised the corresponding statement in the conclusion (lines 308-309).

**Comment 2:** In addition, the potential for residual confounding should be considered, given the substantial differences between occupational groups, including socioeconomic status and work-related stress. Possible misclassification bias may also exist due to the classification of sedentary behavior based solely on occupational categories.

**Response:** Thank you for this comment. We have added potential differences between the two groups, such as socioeconomic status and occupational stress, to the limitations section (lines 292-295).

**Comment 3:** Furthermore, there is an inconsistency regarding the version of the Chinese diabetes guidelines cited for the definition of metabolic syndrome in the study, which should be corrected.

**Response:** Thank you for your comment. We have updated the definition of metabolic syndrome in the abstract to “Guidelines for the Prevention and Treatment of Diabetes Mellitus in China (2024 Edition)” (lines 32-33) and have also revised the main text (lines 103-104).

Reviewer #2

**Comment 1:** A primary concern is the cross-sectional study design, which limits causal inference; the authors must ensure the discussion consistently interprets findings as associations rather than directional relationships. Regarding sampling and selection bias, the recruitment strategy needs a more detailed explanation, particularly acknowledging how online or group-based recruitment impacts the sample's representativeness of the broader population. Furthermore, the validity of the survey instrument must be addressed by describing any pilot testing or reliability assessments, or by explicitly acknowledging the lack of formal validation as a study limitation.

**Response:** We sincerely appreciate your comments. We have replaced all causal expressions in the discussion with statements of association (lines 277-279). In addition, we have added detailed information on sample selection and grouping criteria (lines 87-97). The questionnaire used in this study has been applied in previous surveys of bus drivers and has shown good reliability and validity (lines 116-117).

**Comment 2:** The statistical analysis section also requires further technical detail. The authors should clarify their regression modeling strategy, specifically outlining variable selection criteria, confounder adjustment, reference categories, multicollinearity assessments, and model diagnostics to ensure all relevant confounders were addressed. The authors must ensure that raw anonymized data are accessible, either as supplementary material or via a public repository. Finally, the limitations section should be expanded to include recall bias, residual confounding, and the reliance on self-reported diagnoses without objective biomarkers. Minor improvements are also needed to ensure consistency between percentages and raw numbers, refine the formatting of p-values and confidence intervals, and streamline the discussion to improve flow and reduce repetition.

**Response:** Thank you for your comments. Covariates were selected based on previous literature (lines 117-118). Factors adjusted in the regression model included age, marital status, education level, smoking status, alcohol consumption, length of employment, and weekly working hours, with the non-sedentary group as the reference (lines 133-142). Multicollinearity diagnostics were performed, and all variance inflation factors were below 2 (lines 176-177). In addition, we have re‑uploaded the cleaned and anonymized dataset as supplementary material. We have added recall bias and self-report bias to the limitations (lines 291-292). We have also checked all numerical values and formatting in the tables and streamlined the discussion to reduce repetition.

Reviewer #3

**Comment 1:** The abstract concludes that “Occupational sedentary behavior in males is associated with higher prevalence of metabolic syndrome...”. However, this is demonstrably false based on the study’s own data. In the results section of the abstract, the authors report an Odds Ratio for MetS of 1.05 (95%CI: 0.85-1.29). Since the 95% confidence interval crosses 1.0, this is not a statistically significant finding.

**Response:** Thank you for your comment. We have revised the description in the abstract to state that there is no statistically significant association between occupational sedentary behavior and metabolic syndrome (lines 39-41) and have corrected the corresponding statements in the results section.

**Comment 2:** The abstract must be rewritten to accurately reflect that occupational sedentary behavior was associated with specific components of metabolic syndrome (hypertriglyceridemia, hypoalphalipoproteinemia, and central obesity), but not overall MetS itself.

**Response:** Thank you for your comment. We have revised the relationship between occupational sedentary behavior and overall metabolic syndrome and retained the statistically significant associations with hypertriglyceridemia, hypoalphalipoproteinemia, and central obesity (lines 41-44).

**Comment 3:** The introduction effectively sets up the premise, noting that occupational sedentary behavior is distinct from leisure-time sedentary behavior because it is mandatory and uncontrollable.

**Response:** Thank you for your comment. We have added the rationale for participant grouping in the introduction.

**Comment 4:** The authors should briefly introduce why bus drivers and sanitation workers were specifically chosen as the proxies for sedentary and non-sedentary groups, as these two professions entail vastly different environmental and stress exposures.

**Response:** Thank you for your comment. We have added the rationale for participant selection in the introduction: bus drivers were classified as the occupational sedentary group due to prolonged sitting during work, while sanitation workers were classified as the non‑sedentary group due to prolonged standing and physical activity (lines 75-78).

**Comment 5:** The fundamental flaw in the study design is comparing bus drivers (sedentary) to sanitation workers (non-sedentary). These two groups differ in many ways beyond just sitting time. The authors must acknowledge that bus driving involves high psychological stress, shift work, and vibration, while sanitation work involves heavy physical exertion and high exposure to outdoor pollutants.

**Response:** Thank you for your comment. We have added the inherent differences between the two occupational groups to the limitations: “Due to the substantial differences in work environments, occupational stress, and environmental exposures between the two occupational groups, the study only identifies occupational sedentariness as a potential contributing factor, and future studies are needed to further validate these findings.” (lines 302-306)

**Comment 6:** While the authors adjusted for age, ethnicity, marital status, education level, smoking status, drinking status, length of work, and weekly working hours, they failed to measure or adjust for two massive confounders: Caloric intake and dietary habits are primary drivers of MetS.

**Response:** Thank you for your comment. We have added unmeasured confounders including caloric intake and dietary habits to the limitations: “despite adjustments for confounding factors, residual confounding may persist due to inherent differences in socioeconomic status, work pressure, and lifestyle factors (e.g., diet, non-occupational physical activity) across occupational groups” (lines 292-295).

**Comment 7:** A sanitation worker might rest after work, while a bus driver might exercise. Without adjusting for non-occupational physical activity, isolating the effect of occupational sedentariness is highly problematic.

**Response:** Thank you for your comment. We have addressed the lack of adjustment for non‑occupational physical activity in the limitations: “despite adjustments for confounding factors, residual confounding may persist due to inherent differences in socioeconomic status, work pressure, and lifestyle factors (e.g., diet, non-occupational physical activity) across occupational groups” (lines 292-295).

**Comment 8:** The data shows that the sedentary group had a lower prevalence of hypertension (44.1% vs.54.6%) and hyperglycemia (23.8% vs. 29.4%) compared to the non-sedentary group. In the main text, the authors write: Interestingly, despite no significant difference was observed between the groups (P=0.681), the trend of a higher prevalence of MetS in occupational sedentary group. Reporting a non-significant result as a trend of higher prevalence is statistically inappropriate and must be removed.

**Response:** Thank you for your comment. We have revised the statement to: “The results showed that no significant correlations were observed between occupational sedentary and MetS (*P* > 0.05)” (lines 177-179).

**Comment 10:** Table 3 confirms that MetS is not significantly associated with sedentary behavior across Model 1 (p=0.681), Model 2 (p=0.196), or Model 3 (p=0.285). The manuscript must be revised overall to remove claims that a direct association with overall MetS was found.

**Response:** Thank you for your comment. We have revised all claims suggesting a significant association between occupational sedentary behavior and overall metabolic syndrome.

**Comment 11:** The authors attempt to explain the lower rates of hypertension and hyperglycemia in bus drivers by citing workplace regulations namely, that bus drivers undergo strict blood pressure monitoring and those failing to meet standards are prohibited from driving. This is a textbook example of the Healthy Worker Survivor Effect. Because sick bus drivers are removed from the population, the remaining drivers appear artificially healthier in these metrics. This severely limits the validity of comparing these two specific cohorts and must be explicitly detailed as a major limitation.

**Response:** Thank you for your comment. We have added the Healthy Worker Survivor Effect as a key limitation: “Fourthly, the comparison between bus drivers and sanitation workers may be affected by the Healthy Worker Survivor Effect, which may artificially lower the prevalence of hypertension and hyperglycemia in the remaining driver cohort. Future studies should further explore the prevalence of hypertension and hyperglycemia in other occupational sedentary populations.” (lines 295-299)

**Comment 12:** The discussion regarding skeletal muscle activity, lipoprotein lipase (LPL), and interleukin-6 (IL-6) is well-supported and provides a good biological mechanism for the lipid abnormalities observed. The limitations section currently notes the cross-sectional design and self-reported questionnaire variables. It must be expanded to explicitly state the unmeasured confounders (diet, non-work physical activity) and the immense baseline differences between the specific occupations chosen.

**Response:** Thank you for your comment. We have added the following points to the limitations: (1) “despite adjustments for confounding factors, residual confounding may persist due to inherent differences in socioeconomic status, work pressure, and lifestyle factors (e.g., diet, non-occupational physical activity) across occupational groups” (lines 292-295); (2) “Due to the substantial differences in work environments, occupational stress, and environmental exposures between the two occupational groups, the study only identifies occupational sedentariness as a potential contributing factor, and future studies are needed to further validate these findings” (lines 302-306).

**Suggested citations:**

• Sarfraz, M., Sajid, S., & Ashraf, M. A. (2016). Prevalence and pattern of dyslipidemia in hyperglycemic patients and its associated factors among Pakistani population. Saudi journal of biological sciences, 23(6), 761-766.

• Abbas, G., Salman, A., Rahman, S. U., Ateeq, M. K., Usman, M., Sajid, S., ... & Younas, T. (2017). Aging mechanisms: linking oxidative stress, obesity and inflammation. Matrix Sci. Med, 1(1), 30-33.

• Toor, I. F., Sajid, M., & Sajid, H. U. (2024). Molecular Mechanisms of Diabetes Mellitus. Fundamentals of Cellular and Molecular Biology, 156-176.

**Response:** We have integrated the suggested references into the discussion:

(1) Sarfraz et al. (2016) was cited in the section on the prevalence of hypertriglyceridemia and hyperglycemia (lines 235-238);

(2) Abbas et al. (2017) was cited to explain the mechanism underlying the interaction between age (>55 years) and central obesity (lines 279-283);

(3) Toor et al. (2024) was cited in the section on the long‑term consequences of hyperglycemia and metabolic syndrome (lines 248-251).
