# Supplementary material for: Association between occupational sedentary behavior and metabolic syndrome and related diseases in males: A cross-sectional study
Source: PLoS One. 2026 Jun 26;21(6):e0350342. doi: 10.1371/journal.pone.0350342 (PMC13308799; doi:10.1371/journal.pone.0350342)
Supplement: Peer review history

# Peer review: Decision Letter 0

**DOI:** 10.1371/journal.pone.0350342.r001

**Authors:** Sanaullah Sajid

2 Mar 2026

PONE-D-25-67473

Association between occupational sedentary behavior and metabolic syndrome and related diseases in males: A cross-sectional study

PLOS One

Dear Dr. Shaofan Weng,

Thank you for submitting your manuscript to PLOS ONE. After careful consideration, we feel that it has merit but does not fully meet PLOS ONE’s publication criteria as it currently stands. Therefore, we invite you to submit a revised version of the manuscript that addresses the points raised during the review process.

Please submit your revised manuscript by Apr 16 2026 11:59PM. If you will need more time than this to complete your revisions, please reply to this message or contact the journal office at plosone@plos.org. When you're ready to submit your revision, log on to https://www.editorialmanager.com/pone/ and select the 'Submissions Needing Revision' folder to locate your manuscript file.

Please include the following items when submitting your revised manuscript:

- A letter that responds to each point raised by the academic editor and reviewer(s). You should upload this letter as a separate file labeled 'Response to Reviewers'.
- A marked-up copy of your manuscript that highlights changes made to the original version. You should upload this as a separate file labeled 'Revised Manuscript with Track Changes'.
- An unmarked version of your revised paper without tracked changes. You should upload this as a separate file labeled 'Manuscript'.

If you would like to make changes to your financial disclosure, please include your updated statement in your cover letter. Guidelines for resubmitting your figure files are available below the reviewer comments at the end of this letter.

If applicable, we recommend that you deposit your laboratory protocols in protocols.io to enhance the reproducibility of your results. Protocols.io assigns your protocol its own identifier (DOI) so that it can be cited independently in the future. For instructions see: https://journals.plos.org/plosone/s/submission-guidelines#loc-laboratory-protocols. Additionally, PLOS ONE offers an option for publishing peer-reviewed Lab Protocol articles, which describe protocols hosted on protocols.io. Read more information on sharing protocols at https://plos.org/protocols?utm_medium=editorial-email&utm_source=authorletters&utm_campaign=protocols.

We look forward to receiving your revised manuscript.

Kind regards,

Sanaullah Sajid, Ph.D.

Academic Editor

PLOS One

Journal Requirements:

When submitting your revision, we need you to address these additional requirements.

1. Please ensure that your manuscript meets PLOS ONE's style requirements, including those for file naming. The PLOS ONE style templates can be found at

https://journals.plos.org/plosone/s/file?id=wjVg/PLOSOne_formatting_sample_main_body.pdf and

https://journals.plos.org/plosone/s/file?id=ba62/PLOSOne_formatting_sample_title_authors_affiliations.pdf.

2. Thank you for stating in your Funding Statement:

“This work was supported by the National Key R&D Program of China [grant number

2023YFC2509300], and the Science and Technology Planning Project of Shenzhen

Municipality [grant number KCXFZ20201221173602007].”

Please provide an amended statement that declares *all* the funding or sources of support (whether external or internal to your organization) received during this study, as detailed online in our guide for authors at http://journals.plos.org/plosone/s/submit-now.  Please also include the statement “There was no additional external funding received for this study.” in your updated Funding Statement.

Please include your amended Funding Statement within your cover letter. We will change the online submission form on your behalf.

3. Thank you for stating the following financial disclosure:

“This work was supported by the National Key R&D Program of China [grant number

2023YFC2509300], and the Science and Technology Planning Project of Shenzhen

Municipality [grant number KCXFZ20201221173602007].”

Please state what role the funders took in the study.  If the funders had no role, please state: "The funders had no role in study design, data collection and analysis, decision to publish, or preparation of the manuscript."

If this statement is not correct you must amend it as needed.

Please include this amended Role of Funder statement in your cover letter; we will change the online submission form on your behalf.

4. We note that there is identifying data in the Supporting Information file <Relevant Data>. Due to the inclusion of these potentially identifying data, we have removed this file from your file inventory. Prior to sharing human research participant data, authors should consult with an ethics committee to ensure data are shared in accordance with participant consent and all applicable local laws.

Data sharing should never compromise participant privacy. It is therefore not appropriate to publicly share personally identifiable data on human research participants. The following are examples of data that should not be shared:

-Name, initials, physical address

-Ages more specific than whole numbers

-Internet protocol (IP) address

-Specific dates (birth dates, death dates, examination dates, etc.)

-Contact information such as phone number or email address

-Location data

-ID numbers that seem specific (long numbers, include initials, titled “Hospital ID”) rather than random (small numbers in numerical order)

Data that are not directly identifying may also be inappropriate to share, as in combination they can become identifying. For example, data collected from a small group of participants, vulnerable populations, or private groups should not be shared if they involve indirect identifiers (such as sex, ethnicity, location, etc.) that may risk the identification of study participants.

Additional guidance on preparing raw data for publication can be found in our Data Policy (https://journals.plos.org/plosone/s/data-availability#loc-human-research-participant-data-and-other-sensitive-data) and in the following article: http://www.bmj.com/content/340/bmj.c181.long.

Please remove or anonymize all personal information (<specific identifying information in file to be removed>), ensure that the data shared are in accordance with participant consent, and re-upload a fully anonymized data set. Please note that spreadsheet columns with personal information must be removed and not hidden as all hidden columns will appear in the published file.

5.If the reviewer comments include a recommendation to cite specific previously published works, please review and evaluate these publications to determine whether they are relevant and should be cited. There is no requirement to cite these works unless the editor has indicated otherwise.

Additional Editor Comments (if provided):

Here are the missing comments from Reviewer 3.

While the study utilizes a robust sample size of 2,055 male workers, there are critical flaws in how the data is interpreted and reported specifically a direct contradiction between the statistical results and the abstract's conclusions.

1. The abstract concludes that “Occupational sedentary behavior in males is associated with higher prevalence of metabolic syndrome...”. However, this is demonstrably false based on the study’s own data. In the results section of the abstract, the authors report an Odds Ratio for MetS of 1.05 (95%CI: 0.85-1.29). Since the 95% confidence interval crosses 1.0, this is not a statistically significant finding.

2. The abstract must be rewritten to accurately reflect that occupational sedentary behavior was associated with specific components of metabolic syndrome (hypertriglyceridemia, hypoalphalipoproteinemia, and central obesity), but not overall MetS itself.

3. The introduction effectively sets up the premise, noting that occupational sedentary behavior is distinct from leisure-time sedentary behavior because it is mandatory and uncontrollable.

4. The authors should briefly introduce why bus drivers and sanitation workers were specifically chosen as the proxies for sedentary and non-sedentary groups, as these two professions entail vastly different environmental and stress exposures.

5. The fundamental flaw in the study design is comparing bus drivers (sedentary) to sanitation workers (non-sedentary). These two groups differ in many ways beyond just sitting time. The authors must acknowledge that bus driving involves high psychological stress, shift work, and vibration, while sanitation work involves heavy physical exertion and high exposure to outdoor pollutants.

6. While the authors adjusted for age, ethnicity, marital status, education level, smoking status, drinking status, length of work, and weekly working hours, they failed to measure or adjust for two massive confounders:

7. Caloric intake and dietary habits are primary drivers of MetS.

8. A sanitation worker might rest after work, while a bus driver might exercise. Without adjusting for non-occupational physical activity, isolating the effect of occupational sedentariness is highly problematic.

9. The data shows that the sedentary group had a lower prevalence of hypertension (44.1% vs. 54.6%) and hyperglycemia (23.8% vs. 29.4%) compared to the non-sedentary group. In the main text, the authors write: Interestingly, despite no significant difference was observed between the groups (P=0.681), the trend of a higher prevalence of MetS in occupational

sedentary group.. Reporting a non-significant result as a trend of higher prevalence is statistically inappropriate and must be removed. 10. Table 3 confirms that MetS is not significantly associated with sedentary behavior across Model 1 (p=0.681), Model 2 (p=0.196), or Model 3 (p=0.285). The manuscript must be revised

overall to remove claims that a direct association with overall MetS was found.

11. The authors attempt to explain the lower rates of hypertension and hyperglycemia in bus drivers by citing workplace regulations namely, that bus drivers undergo strict blood pressure monitoring and those failing to meet standards are prohibited from driving. This is a textbook example of the Healthy Worker Survivor Effect. Because sick bus drivers are removed from the population, the remaining drivers appear artificially healthier in these metrics. This severely limits the validity of comparing these two specific cohorts and must be explicitly detailed as a major limitation.

12. The discussion regarding skeletal muscle activity, lipoprotein lipase (LPL), and interleukin-6 (IL-6) is well-supported and provides a good biological mechanism for the lipid abnormalities observed. The limitations section currently notes the cross-sectional design and self-reported questionnaire variables. It must be expanded to explicitly state the unmeasured confounders (diet, non-work physical activity) and the immense baseline differences between the specific occupations chosen.

There are a few papers that align with the physiological and metabolic aspects of the manuscript. We suggest to appropriately integrate them:

• Sarfraz, M., Sajid, S., & Ashraf, M. A. (2016). Prevalence and pattern of dyslipidemia in hyperglycemic patients and its associated factors among Pakistani population. Saudi journal of biological sciences, 23(6), 761-766.

Where: In the Discussion section. The authors discuss the prevalence of hypertriglyceridemia and hyperglycemia in their cohorts. You use this paper to contextualize the regional prevalence of dyslipidemia and how it clusters with hyperglycemia in Asian populations.

• Abbas, G., Salman, A., Rahman, S. U., Ateeq, M. K., Usman, M., Sajid, S., ... & Younas, T. (2017). Aging mechanisms: linking oxidative stress, obesity and inflammation. Matrix Sci. Med, 1(1), 30-33.

Where: In the Discussion section regarding the subgroup analysis. The authors note a significant interaction between age (>55 years) and central obesity. You use this paper to help

them explain the biological mechanisms

, specifically how aging and inflammation exacerbate obesity-related metabolic dysfunction

• Toor, I. F., Sajid, M., & Sajid, H. U. (2024). Molecular Mechanisms of Diabetes Mellitus. Fundamentals of Cellular and Molecular Biology, 156-176.

Where: In the Introduction or Discussion. The authors should expand on the long-term consequences of the hyperglycemia and metabolic syndrome observed in their study.

These recent publications could serve as up-to-date references on the molecular progression and management of diabetes.

[Note: HTML markup is below. Please do not edit.]

Reviewers' comments:

Reviewer's Responses to Questions

**Comments to the Author**

1. Is the manuscript technically sound, and do the data support the conclusions?

The manuscript must describe a technically sound piece of scientific research with data that supports the conclusions. Experiments must have been conducted rigorously, with appropriate controls, replication, and sample sizes. The conclusions must be drawn appropriately based on the data presented.

Reviewer #1: Yes

Reviewer #2: Partly

Reviewer #3: Partly

**********

2. Has the statistical analysis been performed appropriately and rigorously?

Reviewer #1: No

Reviewer #2: No

Reviewer #3: No

**********

3. Have the authors made all data underlying the findings in their manuscript fully available?

The PLOS Data policy requires authors to make all data underlying the findings described in their manuscript fully available without restriction, with rare exception (please refer to the Data Availability Statement in the manuscript PDF file). The data should be provided as part of the manuscript or its supporting information, or deposited to a public repository. For example, in addition to summary statistics, the data points behind means, medians and variance measures should be available. If there are restrictions on publicly sharing data—e.g. participant privacy or use of data from a third party—those must be specified.

Reviewer #1: Yes

Reviewer #2: No

Reviewer #3: No

**********

4. Is the manuscript presented in an intelligible fashion and written in standard English?

PLOS ONE does not copyedit accepted manuscripts, so the language in submitted articles must be clear, correct, and unambiguous. Any typographical or grammatical errors should be corrected at revision, so please note any specific errors here.

Reviewer #1: Yes

Reviewer #2: Yes

Reviewer #3: No

**********

5. Review Comments to the Author

Please use the space provided to explain your answers to the questions above. You may also include additional comments for the author, including concerns about dual publication, research ethics, or publication ethics. (Please upload your review as an attachment if it exceeds 20,000 characters)

Reviewer #1: -The association between occupational sedentary behavior and metabolic syndrome was not statistically significant; therefore, it is recommended that this relationship be revised accordingly in both the abstract and the conclusion.

In addition, the potential for residual confounding should be considered, given the substantial differences between occupational groups, including socioeconomic status and work-related stress. Possible misclassification bias may also exist due to the classification of sedentary behavior based solely on occupational categories.

Furthermore, there is an inconsistency regarding the version of the Chinese diabetes guidelines cited for the definition of metabolic syndrome in the study, which should be corrected.

Reviewer #2: A primary concern is the cross-sectional study design, which limits causal inference; the authors must ensure the discussion consistently interprets findings as associations rather than directional relationships. Regarding sampling and selection bias, the recruitment strategy needs a more detailed explanation, particularly acknowledging how online or group-based recruitment impacts the sample's representativeness of the broader population. Furthermore, the validity of the survey instrument must be addressed by describing any pilot testing or reliability assessments, or by explicitly acknowledging the lack of formal validation as a study limitation.

The statistical analysis section also requires further technical detail. The authors should clarify their regression modeling strategy, specifically outlining variable selection criteria, confounder adjustment, reference categories, multicollinearity assessments, and model diagnostics to ensure all relevant confounders were addressed. The authors must ensure that raw anonymized data are accessible, either as supplementary material or via a public repository. Finally, the limitations section should be expanded to include recall bias, residual confounding, and the reliance on self-reported diagnoses without objective biomarkers. Minor improvements are also needed to ensure consistency between percentages and raw numbers, refine the formatting of p-values and confidence intervals, and streamline the discussion to improve flow and reduce repetition.

Reviewer #3: While the study utilizes a robust sample size of 2,055 male workers, there are critical flaws in how the data is interpreted and reported specifically a direct contradiction between the statistical results and the abstract's conclusions.

**********

6. PLOS authors have the option to publish the peer review history of their article (what does this mean?). If published, this will include your full peer review and any attached files.

If you choose “no”, your identity will remain anonymous but your review may still be made public.

**Do you want your identity to be public for this peer review?**  For information about this choice, including consent withdrawal, please see our Privacy Policy.

Reviewer #1: No

Reviewer #2: No

Reviewer #3: No

**********

[NOTE: If reviewer comments were submitted as an attachment file, they will be attached to this email and accessible via the submission site. Please log into your account, locate the manuscript record, and check for the action link "View Attachments". If this link does not appear, there are no attachment files.]

To ensure your figures meet our technical requirements, please review our figure guidelines: https://journals.plos.org/plosone/s/figures

You may also use PLOS’s free figure tool, NAAS, to help you prepare publication quality figures: https://journals.plos.org/plosone/s/figures#loc-tools-for-figure-preparation.

NAAS will assess whether your figures meet our technical requirements by comparing each figure against our figure specifications.

Attachment Submitted filename: Comments to Author.pdf

Attachment Submitted filename: Recommendation.pdf

# Author comment: Author response to Decision Letter 1

**DOI:** 10.1371/journal.pone.0350342.r002

30 Apr 2026

We have carefully addressed all the comments and suggestions from the reviewers and editors in the revised manuscript. A detailed point-by-point response letter is provided as an attachment, outlining how each comment has been addressed and the corresponding changes made in the manuscript.

Attachment Submitted filename: Response to Reviewers.docx

# Editor report: Decision Letter 1

**DOI:** 10.1371/journal.pone.0350342.r003

**Authors:** Sanaullah Sajid

13 May 2026

Association between occupational sedentary behavior and metabolic syndrome and related diseases in males: A cross-sectional study

PONE-D-25-67473R1

Dear Dr. Weng,

We’re pleased to inform you that your manuscript has been judged scientifically suitable for publication and will be formally accepted for publication once it meets all outstanding technical requirements.

Within one week, you’ll receive an e-mail detailing the required amendments. When these have been addressed, you’ll receive a formal acceptance letter and your manuscript will be scheduled for publication.

An invoice will be generated when your article is formally accepted. Please note, if your institution has a publishing partnership with PLOS and your article meets the relevant criteria, all or part of your publication costs will be covered. Please make sure your user information is up-to-date by logging into Editorial Manager at Editorial Manager® and clicking the ‘Update My Information' link at the top of the page. For questions related to billing, please contact billing support.

If your institution or institutions have a press office, please notify them about your upcoming paper to help maximize its impact. If they’ll be preparing press materials, please inform our press team as soon as possible -- no later than 48 hours after receiving the formal acceptance. Your manuscript will remain under strict press embargo until 2 pm Eastern Time on the date of publication. For more information, please contact onepress@plos.org.

Kind regards,

Sanaullah Sajid, Ph.D.

Academic Editor

PLOS One

Additional Editor Comments (optional):

Reviewers' comments:

# Editor report: Acceptance letter

**DOI:** 10.1371/journal.pone.0350342.r004

**Authors:** Sanaullah Sajid

PONE-D-25-67473R1

PLOS One

Dear Dr. Weng,

I'm pleased to inform you that your manuscript has been deemed suitable for publication in PLOS One. Congratulations! Your manuscript is now being handed over to our production team.

At this stage, our production department will prepare your paper for publication. This includes ensuring the following:

* All references, tables, and figures are properly cited

* All relevant supporting information is included in the manuscript submission,

* There are no issues that prevent the paper from being properly typeset

You will receive further instructions from the production team, including instructions on how to review your proof when it is ready. Please keep in mind that we are working through a large volume of accepted articles, so please give us a few days to review your paper and let you know the next and final steps.

Lastly, if your institution or institutions have a press office, please let them know about your upcoming paper now to help maximize its impact. If they'll be preparing press materials, please inform our press team within the next 48 hours. Your manuscript will remain under strict press embargo until 2 pm Eastern Time on the date of publication. For more information, please contact onepress@plos.org.

You will receive an invoice from PLOS for your publication fee after your manuscript has reached the completed accept phase. If you receive an email requesting payment before acceptance or for any other service, this may be a phishing scheme. Learn how to identify phishing emails and protect your accounts at https://explore.plos.org/phishing.

If we can help with anything else, please email us at customercare@plos.org.

Thank you for submitting your work to PLOS ONE and supporting open access.

Kind regards,

PLOS ONE Editorial Office Staff

on behalf of

Dr. Sanaullah Sajid

Academic Editor

PLOS One
